# Prolactinomas treated with dopaminergic agonists: behavior in different moments of life

**DOI:** 10.61622/rbgo/2026rbgo9

**Published:** 2026-04-17

**Authors:** Marcela Souza Carneiro, Heraldo Mendes Garmes, Gabriela Pravatta-Rezende, Ticiana Aparecida Alves de Mira, Daniela Angerame Yela, Cristina Laguna Benetti-Pinto

**Affiliations:** 1 Universidade Estadual de Campinas Campinas SP Brazil Universidade Estadual de Campinas, Campinas, SP, Brazil.

**Keywords:** Prolactin, Hyperprolactinemia, Prolactinoma, Dopamine agonists, Pregnancy, Menopause

## Abstract

**Objective:**

To evaluate the behavior of pituitary adenomas following cessation of cabergoline (CAB) treatment, postpartum, and postmenopause, in comparison to idiopathic hyperprolactinemia (HPRL).

**Methods:**

This retrospective cohort study included women with HPRL treated with CAB under three conditions: treatment for at least two years and follow-up for a minimum of one year after treatment discontinuation, postpartum, and postmenopause. The evaluated parameters included tumor size, prolactin (PRL) levels, and clinical symptomatology.

**Results:**

A total of 160 women with idiopathic HPRL, micro- or macroadenomas, with symptom onset at 29.3±9.0 years, were included. Menstrual alterations occurred in 70% and galactorrhea in 63% of cases. PRL normalization occurred more rapidly in idiopathic HPRL than in tumors. However, symptom resolution required a similar time across all groups. All macroadenomas regressed to <10 mm. After CAB withdrawal, reintroduction was necessary in all macroadenomas, 53% of microadenomas, and 23% of idiopathic HPRL. Following postpartum CAB withdrawal, mean serum PRL levels increased by 300% in macroadenomas, 218% in microadenomas, and 11% in idiopathic HPRL; approximately 75% of all patients experienced symptom recurrence. Postmenopausal PRL normalization occurred in over 40% of cases. One untreated idiopathic HPRL case developed a microadenoma 12 months after menopause.

**Conclusion:**

HPRL presented a high recurrence rate following CAB discontinuation, regardless of tumor presence or treatment duration, with no reduction in recurrence observed after the postpartum period.

## Introduction

Hyperprolactinemia (HPRL), defined by elevated serum prolactin (PRL) levels, is a relatively common cause of menstrual irregularities, galactorrhea, and infertility in women of reproductive age.^(1,2)^ It is typically diagnosed in the 3rd and 5th decades of life.^(1,3)^ HPRL has multiple causes that can be subdivided into physiological factors (such as pregnancy and breastfeeding); pharmacological factors involving the use of medications, particularly antipsychotics; and pathological causes, including certain chronic diseases or inappropriate PRL secretion from the pituitary gland due to prolactinomas or associated with disconnection of the pituitary stalk due to lesions of the sellar region.^(1-4)^ When no specific cause was identified, the HPRL was considered idiopathic.^(3,4)^ Excluding physiological and pharmacological causes, pituitary adenomas that produce prolactin are the most common etiologies.^(2,5)^ Treatment is commonly clinical and prolonged, often involving dopaminergic agonists (DA).^(2-7)^ Cabergoline (CAB) is currently the preferred first-line DA owing to its convenient dosing regimen, good tolerability, effectiveness in symptom control, restoration of ovarian function, fertility improvement, mitigation of hypoestrogenism-related effects, remission of galactorrhea, tumor size control, and consequent alleviation of compressive effects.^(2-4,6)^ However, the relationship between prolactin secretion and tumor behavior after treatment discontinuation is not well established.

Prolactin secretion is influenced by the menstrual cycle, menopause, and pregnancy. Estrogen, a major ovarian hormone, affects prolactin secretion through various mechanisms, including the regulation of PRL gene expression, downregulation of dopaminergic receptor expression, and stimulation of lactotroph receptors. Thus, estrogen is considered to be a prolactin-releasing factor.^(8-10)^ During pregnancy, a state characterized by elevated circulating estrogen levels, there may be potential stimulation of prolactin production and adenoma growth.^(1)^ In this sense, a study has shown a significative improvement in hyperprolactinemia in the postpartum period. Conversely, during menopause, a reduction in ovarian estrogen production results in the decreased stimulation of prolactin secretion and lactotroph proliferation.^(11-15)^

This study assessed, within a cohort from a single university hospital, the response to treatment with a specific dopaminergic agonist, CAB, in areas where limited evidence is available. Specifically, we examined the behavior of HPRL due to prolactinoma compared with idiopathic HPRL in three scenarios: treatment discontinuation, postpartum, and postmenopause.

## Methods

This retrospective cohort study included women diagnosed with hyperprolactinemia (HPRL) and treated at the Department of Obstetrics and Gynecology of the Faculty of Medical Sciences, University of Campinas (UNICAMP), Brazil—a tertiary referral center—between 2010 January and 2020 December. Eligible participants had a minimum follow-up of three years.

We included women aged 18–40 years with at least two serum prolactin (PRL) values above the reference range and clinical symptoms attributable to HPRL, including menstrual irregularities, galactorrhea, and/or infertility (defined as failure to conceive after ≥12 months of unprotected intercourse). Macroprolactinemia was excluded in all patients using polyethylene glycol (PEG) precipitation assay.

We excluded women with physiological causes of hyperprolactinemia (pregnancy, lactation), medication-induced HPRL (e.g., antipsychotics, antidepressants), illicit drug use, chronic systemic diseases (renal insufficiency, liver cirrhosis, primary hypothyroidism), and pituitary stalk compression or empty sella syndrome. Patients with giant adenomas were also excluded. Thus, the final cohort comprised women with either pituitary adenoma-related HPRL (microadenomas <1 cm or macroadenomas ≥1 cm) or idiopathic HPRL.

All patients underwent imaging of the sella turcica to assess the presence and size of adenomas. Participants were categorized into three groups: microadenoma, macroadenoma, and idiopathic hyperprolactinemia. Group allocation was based on imaging findings.

All women were treated with cabergoline (CAB) at an initial dose of 0.5 mg once weekly. Follow-up evaluations occurred every 8–12 weeks, including reassessment of serum PRL and symptomatology. Dose adjustments of 0.25 mg were made as needed to achieve PRL normalization using the lowest effective dose. Once normalization of symptoms and PRL levels was sustained, treatment was continued for at least two years. After this period, gradual tapering of CAB (by 0.25 mg every 8–12 weeks) was implemented until full withdrawal. Follow-up continued every 3–6 months thereafter.

Women who became pregnant during treatment had CAB discontinued during pregnancy and resumed follow-up after childbirth or lactation. Postmenopausal women discontinued CAB and were followed accordingly.

Variables analyzed included age at symptom onset and diagnosis, PRL, FSH, and TSH levels, time to PRL normalization, time to symptom resolution, tumor size, and reported symptoms. Group comparisons were made among the three etiological categories of HPRL.

Categorical variables were summarized using absolute and relative frequencies and compared using Chi-square or Fisher's exact tests. Numerical variables were described by means, standard deviations, medians, and interquartile ranges, and compared across groups using the Kruskal–Wallis test followed by Dunn's post hoc test. Spearman's correlation coefficient was used to assess relationships between numerical variables. Statistical significance was defined as *p* < 0.05. All analyses were performed using SAS version 9.4 (Cary, NC, USA).

As this was a retrospective study using anonymized data collected from medical records, written informed consent was not required under institutional policy.

The study was approved by the institutional ethics committee (CAAE: 51410221.1.0000.5404; Approval number 5.010.283).

## Results

The medical records of 200 women with idiopathic or secondary prolactinoma-induced HPRL were evaluated. After exclusion due to the absence of imaging, incorrect or non-adherent medication use, or missed follow-up appointments, 160 women diagnosed with HPRL were included in the analysis. They had been treated for at least 2 years and followed up for at least another year post-event under investigation. Among them, 80 were diagnosed with microadenoma (n=61) or macroadenoma (n=19), whereas the remaining 80 had idiopathic HPRL. The mean age at symptom onset was 29.3±9.0 years, and diagnosis occurred at 31.7±9.5 years. The mean body mass index (BMI) was 27.99±6.19 kg/m², with 58% being nulliparous, 27% having up to two pregnancies, and 15% having been pregnant three or more times. At diagnosis, average serum PRL levels were 118.67±116.85 ng/mL, FSH levels were 6.62±7.08 mIU/mL, and TSH levels were 2.77±1.66 mIU/mL (Table 1).

**Table 1:** Variables were analyzed at the time of hyperprolactinemia diagnosis and the first and second reevaluation after initiating cabergoline treatment

Variables		At diagnosis (%)	1^st^ reevaluation (%)	2^nd^ reevaluation (%)
Age at Diagnosis (years)		31.7±9.5	--	--
Age at Symptom Onset (years)		29.3±9.0		
BMI (kg/m²)		27.99±6.19	--	--
Galactorrhea		63	13	10
Menstrual Alteration				
	Prolonged Cycles	33	14	8
	Amenorrhea	37	13	8
Headache		24	15	10
Visual Disturbances		4	1	4
Serum PRL (ng/mL)		118.67±116.85	32.32 ± 39.39	25.68±28.65
Normal Prolactin		---	60	67

Reevaluations conducted every 8 to 12 weeks

Symptoms of galactorrhea, amenorrhea, and prolonged menstrual cycles were reported by 63%, 37%, and 33% of women, respectively (70% of them reported menstrual alterations). Headache and visual disturbances were reported by 24% and 4% of patients, respectively, at the time of diagnosis. Only 17% of the women reported infertility with immediate reproductive desire.

All participants initially received a prescription of one 0.5 mg CAB tablet per week. Considering that the average time for symptoms and serum PRL level re-evaluation was 8–12 weeks, 60% of the women already had serum PRL levels within the normal range at the first re-evaluation, with at least 85% being asymptomatic. In the second re-evaluation, approximately 7 of the 10 women had normalized PRL levels, while at least 9 of the 10 women remained symptom-free (Table 1). The average time for PRL serum level normalization in the total group was 6.64±5.93 months, with a median of 6 months, and for clinical symptom normalization (excluding infertility) it was 6.89±7.01 month.

The maximum CAB dose required for PRL level or symptom normalization was 5 mg/week for only one patient (due to self-administration error leading to higher-than-prescribed dose), between 2 and 3 mg/week for five patients, and for the vast majority (n=154), ranging from 0.25 to 2.0 mg/week.

The analyzed variables were compared according to the cause of HPRL (Table 2). Women with HPRL secondary to macroadenoma of the pituitary gland were younger at symptom onset and had higher PRL levels than those with microadenoma or nontumoral idiopathic HPRL. Normalization of serum PRL levels occurred more rapidly among women with idiopathic HPRL, with no difference observed between women with microadenomas and those with macroadenomas. Clinical recurrence occurred in all macroadenomas (100%), as none were able to maintain suspension.

**Table 2:** Comparative analysis according to the cause of HPRL, considering the response to Cabergoline, treatment withdrawal, occurrence of pregnancy, or menopause

		Microadenoma# (n=61)	Macroadenoma## (n=19)	Idiopathic### (n=80)	p-value *
Age (years)		32.7+9.9	27.7±8.7	32.1±9.2	0.141
Age at Symptom Onset (years)		29.5+9.7	24.4±8.0	30.6±8.3	0.036 (2#3)
TSH (mUI/mL)		2.79+1.93	2.84±1.73	2.75±1.40	0.798
FSH (mUI/mL)		7.05+8.92	5.74±3.49	6.54±6.29	0.994
PRL at Diagnosis (ng/mL)		124.53+106.89	244.91 ±219.89	83.78±50.71	<0.001 2# (1,3)
Tumor Size at Diagnosis (mm)		5.32+1.82	12.79±2.72	—	<0.001 1#2
Time to PRL Normalization (months)		8.1+6.6	7.8±8.1	4.5±3.2	0.024 1#3
Time to Symptom Normalization (months)		6.9+7.3	9.8±13.9	6.0±3.8	0.925
**Withdrawal of treatment (n=44)**		**Microadenoma** # **(n=19)**	**Macroadenoma** ## **(n=3)**	**Idiopathic** ### **(n=22)**	p-value*
PRL at CAB Withdrawal					
	Mean+SD (ng/mL)	15.28+16.31	10.75±2.63	17.53±22.21	
	<25 ng/L(%)	83	100.00	86	0.885
	>25 ng/L (%)	17	—	14	
Tumor Size at Withdrawal (mm)		1.50+2.12	4.0 (all regressed, <10 mm)	---	0.221
Follow-up without Medication (months)		13.0+15.51	4.0±1.73	10.14±7.47	0.220
PRL at Reevaluation without CAB					
	Mean (ng/mL)	47.86+39.22	64.43±70.70	53.13±44.33	
	<25ng/L (%)	28.6	33.3	14.3	0.982
	>25 ng/L (%)	71.4	66.7	85.7	
Tumor Size in Maintained without CAB (n/total)		2.92+2.69 (10/19)	Reintroduced in all (0/3)	—	—
CAB Reintroduction					
Number (n/total)		(9/19)	(3/3)	(5/22)	
Time without CAB (months)		14.8+13.5	---	4.6±1.8	0.182
	Tumor diameter (mm)	4.00	5.33±5.03	8.0 (1/22)	0.529
**Pregnancy (n=18)**		**Microadenoma**# (n=6)	**Macroadenoma** ## **(n=3)**	**Idiopathic** ### **(n=9)**	p-value*
Tumor Size before Pregnancy (mm)		5.35+3.07	8.33±7.37	—	0.040
PRL before Pregnancy (mean, median, Q1, Q3)		27.2+26.66;	78.83±100.91	35.2±43.5	0.646
PRL after Pregnancy (ng/mL)		17.5 (Q1=9.1; Q3=39.9) 59.16+22.53	28.6 (Q1=12.9; Q3=195.0) 236.93±328.2	25.3 (Q1=13.2; Q3=31.5) 38.90±20.94	0.570
Tumor Size after Pregnancy (mm)		No data	10.0 (n=1)	6.0 (n=1)	
Breastfeeding Duration (months)		11.6+7.73	11.0	18.0±19.8	0.796
**Menopause(n=8)**		**Microadenoma** # **(n=5)**	**Macroadenoma** ## **(n=1)**	**Idiopathic** ### **(n=2)**	p-value*
Tumor Size at Menopause (mm)		5.50+3.87	10.0	---	0.526
PRL before Menopause (mean, median, Q1, Q3)		41.29+39.17	1.82	80.36±57.48	0.407
PRL after Menopause (ng/mL)		41.4 (Q1=11.9; Q3=51.0) 42.94+28.73	1.8 (Q1=1.8; Q3=1.8) 7.74	80.4 (Q1=39.7; Q3=121.0) 31.75± 40.26	0.637
Tumor Size after Menopause (mm)		2.33+4.04	7.50	5.0 (n=1 diagnosis of microadenoma 12 months after menopause)	

Chi-square or Fisher's exact tests were used to compare categorical variables among HPRL causes.

* p-values refer to the Kruskal–Wallis test for comparison among the three groups, followed by Dunn's test for multiple comparisons. To interpret the p-values:

# microadenoma,

## macroadenoma, and

### idiopathic.

Considering that the CAB dose was gradually reduced to evaluate complete withdrawal, it was observed that, in most women, symptoms reappeared and serum PRL levels increased. Only 44 women discontinued CAB treatment. The first re-evaluation took place after 10.69 ± 10.91 months. In 80% of the patients, PRL levels were already above the normal range, while 28% reported galactorrhea, 13% reported long menstrual cycles, and 5% reported amenorrhea (thus, 18% had some form of menstrual alteration), and 18% reported headaches. Among women with macroadenoma (n=19), only three maintained good control with decreasing dose of CAB allowing complete withdrawal of CAB. Although, all patients experienced a reduction in the largest tumor diameter (< 1 cm), they experienced symptom recurrence, requiring the reintroduction of medication within a short interval.

**Figure 1:**
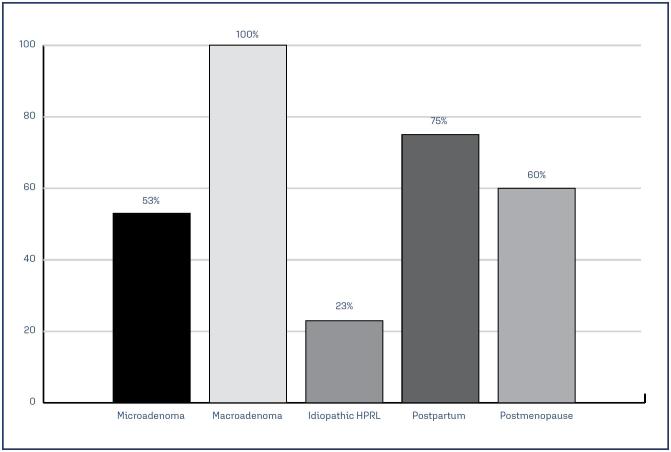
Hyperprolactinemia recurrence after CAB interruption

Among the 18 women who became pregnant while using CAB, six, three, and nine were diagnosed with microadenoma, macroadenoma, and idiopathic HPRL, respectively. At the time of pregnancy, 94% of them were using up to 0.5 mg/week of CAB, and all tumors had subcentimetric measurements. The average serum PRL levels at the last measurement taken before pregnancy were still above the normal range in patients with macroadenomas (Table 2). All patients suspended their medication during pregnancy and there was no reintroduction of the medication during gestation. After childbirth and cessation of breastfeeding, 12 women returned for evaluation, with 75% already showing increased serum PRL levels. Regarding symptoms, galactorrhea was reported by 50%, and long menstrual cycles by 25% (this number might be underestimated, as some were using combined hormonal contraceptives). None of the women reported amenorrhea or visual disturbances, whereas half reported headaches (Figure 1).

During the extended follow-up of 160 women, 5% (n=8) reached menopause, with 5 in the microadenoma group, one in the macroadenoma group, and two in the idiopathic HPRL group. All patients suspended their medications at the time of menopausal diagnosis. PRL levels before and after menopause did not differ between the groups according to the cause. None of the women reported galactorrhea, headaches, or visual disturbances during post-menopause evaluation (Table 2). However, one of the women with idiopathic hyperprolactinemia was diagnosed with a microadenoma 12 months after discontinuing CAB, without the need to reintroduce CAB at this moment. Spearman's correlation analysis, including the analyzed variables, showed a positive correlation only between serum PRL level at diagnosis and the largest tumor diameter. A larger tumor diameter was associated with higher serum PRL levels (R=0.292, p=0.0085). The other clinical and laboratory variables analyzed did not correlate with PRL levels or tumor diameter.

## Discussion

This study presents a review of cases of HPRL treated exclusively with CAB and without prior surgical treatment, with a follow-up period of at least 3 years, in a single center. Three situations with gaps in knowledge were examined: the behavior of prolactin secretion and tumors after withdrawal from DA treatment, pregnancy, and menopause. The main results reaffirmed that CAB quickly controlled the symptoms and PRL levels. Of the patients who had been taking the medication regularly for at least 24 months and attempted the dose-reduction withdrawal regimen, only approximately 30% were able to completely discontinue the medication. Of these, three patients with macroprolactinoma, 47.3% of the patients with microprolactinoma, and only 22.7% of the patients with idiopathic hyperprolactinemia had recurrence of symptoms and elevated PRL levels, requiring reintroduction of CAB. After pregnancy and lactation, 75% of the women required reintroduction of DA due to the presence of symptoms and elevated PRL levels, but 25% did not. After menopause, even with discontinuation of CAB, PRL levels normalized in more than 40% of the women, and the others, although presenting levels slightly above the upper limit, did not present an increase in symptoms.

The objectives of DA treatment for HPRL are to restore eugonadism and fertility, stop galactorrhea, and control tumor growth. Once these objectives are achieved, there are many uncertainties regarding the manner and timing of medication withdrawal and duration of symptom remission.^(16)^ Our results demonstrate that the response to CAB is rapid, effectively controlling symptoms, PRL levels, and reducing tumor size relatively quickly. Despite the potential cost savings from treatment discontinuation (possible in 30% of women with HPRL due to microadenoma or idiopathic causes and in 15% with macroadenoma), it should be emphasized that within a short period, approximately 80% of women had PRL levels above normal, and at least 50% were symptomatic, requiring reintroduction of treatment.

When women become pregnant, those with noncompressive microadenomas or macroadenomas typically discontinue treatment. Theoretically, prolactinomas may increase in size during pregnancy because of the stimulatory effect of high estrogen levels and treatment discontinuation. For microadenomas, the risk of symptomatic enlargement is relatively low, occurring in 2%–3% of cases, but the risk is higher for macroadenomas (25–30%). Breastfeeding can stimulate PRL secretion but does not seem to increase tumor size. Some evidence suggests that well-controlled micro-or macroadenomas before pregnancy can experience prolonged remission after pregnancy in up to 40% of cases; however, such evidence remains limited.^(17-19)^ The lack of systematic post-pregnancy imaging data for most women with prolactin-secreting microadenomas and macroadenomas, even though all had subcentimetric measurements before pregnancy; this gap limits the ability to accurately determine the true risk of tumor enlargement in the postpartum period, as highlighted by previous studies showing that the risk of clinically relevant growth, although generally low, cannot be excluded without adequate follow-up.

In our case series, 50% of the 18 pregnant women had prolactinomas, and at the time of pregnancy, all macroadenomas measured <1 cm. After pregnancy and lactation, 75% of the women had increased PRL levels and the majority had symptoms. Therefore, our results showed that 25% of the patients were in remission, providing more data for understanding the behavior of HPRL after pregnancy.

After menopause, a state of permanent hypoestrogenism exists, indicating a potential influence on the treatment of prolactinomas.^(11-15)^ Considering the state of physiological hypoestrogenism, asymptomatic postmenopausal women diagnosed with a microadenoma or idiopathic hyperprolactinemia during their reproductive life may not require treatment with a dopaminergic agonist. In selected cases of macroprolactinoma with a significant reduction in volume and no apparent risk of optic chiasm compression, withdrawal of DA treatment is suggested while carefully monitoring the tumor. In contrast, macroprolactinomas with a compressive risk may require maintenance of medication.^(20)^

It is important to emphasize that in women, most prolactinomas are diagnosed during the reproductive period. The diagnosis in postmenopausal women is less common and generally occurs with a significant delay. Prospective data on the management of prolactinomas outside of the reproductive period are scarce. The literature shows that 40%–60% of cases treated with AD show good control after discontinuing medication. HPLR remission after withdrawal occurs mainly in women with microprolactinomas. The recurrence rate varies, ranging from approximately 7% to 33% of patients.^(21-24)^

In our case series, the number of women who reached menopause was low. However, considering that approximately 60% of them had PRL levels above normal values at the evaluation before menopause and that the treatment was discontinued at menopause, there was a reduction in PRL levels even with the withdrawal of CAB. PRL levels remained within the normal range in 50% of the cases, and all women were asymptomatic without galactorrhea or compressive symptoms. However, even patients with idiopathic HPRL require monitoring, as we identified a case of tumor appearance after discontinuation of CAB.

The primary limitation of the study is the small number of women followed up after menopause or pregnancy, which limits the conclusions. Nevertheless, recommendations in the literature for managing menopause and pregnancy are generally based on case series described by the authors with data from single or multiple centers, as well as review studies. Also, the exclusion of women with incorrect or non-adherent medication use, or missed follow-up appointments, with selection of highly adherent patients might limit the generalizability of the results to a broader population. However, a strong point of the study is that it included a cohort of 160 women, all followed in a single center, with a well-defined protocol over a long period, and with a considerable number of women evaluated after CAB discontinuation.

## Conclusion

Women with hyperprolactinemia due to prolactinoma or idiopathic causes have a high prevalence of galactorrhea and menstrual cycle disturbances. Symptoms and PRL levels normalized after approximately 6 months of CAB treatment, confirming the remarkable efficacy of this dopamine agonist. However, treatment discontinuation is not feasible in many patients. After CAB discontinuation, most patients who met the discontinuation goals did not experienced an increase in PRL levels, and approximately one-third redeveloped symptoms. After pregnancy and lactation, one-quarter of patients did not need to reintroduce medication. After menopause, discontinuation of dopamine agonists is possible, but ongoing monitoring is necessary regardless of symptoms. During the reproductive phase of life, hyperprolactinemia has a high recurrence rate, but attempts to discontinue CAB should be encouraged, especially in cases of microadenomas, with long-term PRL normalization and without symptoms for a prolonged period.

## Data Availability

The research data are described in the article presented.
